# Promoting Health Equity: Identifying Parent and Child Reactions to a Culturally-Grounded Obesity Prevention Program Specifically Designed for Black Girls Using Community-Engaged Research

**DOI:** 10.3390/children10030417

**Published:** 2023-02-21

**Authors:** Haley Allen, Chishinga Callender, Debbe Thompson

**Affiliations:** 1 USDA/ARS Children’s Nutrition Research Center, Department of Pediatrics, Baylor College of Medicine, Houston, TX 77030, USA; 2 Department of Natural Science, Rice University, Houston, TX 77005, USA

**Keywords:** obesity prevention, community-engaged research, health equity

## Abstract

The Butterfly Girls (BFG) Study is a culturally and developmentally appropriate online obesity prevention program for 8–10-year-old Black girls designed with key stakeholders in the Black community. This multi-methods investigation, conducted with parent–child dyads who participated in an outcome evaluation of the intervention, aimed to understand parent and child reactions to the program. We were particularly interested in understanding perceptions regarding its cultural and developmental appropriateness, relevance and acceptability. Program participation and survey data (demographics, parent and child write-in comments on process evaluation surveys) were analyzed. Participation data demonstrated high adherence in treatment and comparison groups. Descriptive statistics were calculated for survey data and highlighted the socioeconomic diversity of the sample. Post intervention surveys included two fill-in-the-blank questions for parents (n = 184 for question 1, n = 65 for question 2) and one for children (n = 32). Comments were analyzed using structured thematic analysis. The majority of the feedback from child participants was complimentary and many found the program relatable. Among the parent responses, the majority found the program to be beneficial in its educational nature and in promoting behavior change. This multi-methods analysis suggests that the BFG program was perceived as beneficial by parents while being culturally and developmentally appropriate and engaging for young Black girls, highlighting the importance of co-collaboration in program development.

## 1. Introduction

Obesity prevalence in the United States is at an all-time high. Between 2017–2020, obesity prevalence among 2–19-year-olds was 19.7% [1]; in 2020, it had increased to 22.4% [2]. Prevalence is not equally distributed. Compared to their non-Hispanic White peers (14.8%), Black girls (29.1%) are more likely to have obesity [3]. This disparity is multi-factorial as Black communities are more likely to be exposed to social determinants of health that increase their risk of developing obesity [4]. For instance, hyper-palatable, low-nutrient foods are more likely to be marketed to Black Americans [5]. In addition, excess weight may be perceived differently across ethnic groups [6]. 

To effectively address disparities and reduce obesity risk, convenient, accessible and personally relevant interventions are needed. Online interventions offer one possible route of providing convenience and accessibility given that internet access has increased for all racial and ethnic groups [7] and usage among youth is high [8]. Few online interventions promoting healthy diet and physical activity behaviors have been specifically developed for pre-adolescent Black children, or girls, specifically [9,10,11,12,13]. Furthermore, findings from obesity prevention programs for pre-adolescent Black girls highlight the importance of culturally tailored programming [14] as culture influences beliefs and practices around health habits and body image [15]. Finally, given that obese youth are more likely to become obese adults [16], developing effective interventions for youth at the greatest risk of having obesity is of paramount importance. 

Addressing obesity-related disparities in Black girls is a key aspect of designing programs to achieve health equity. Health equity is evident when everyone is equally positioned to be as healthy as possible [17]. Ensuring that everyone has a fair and just opportunity to achieve optimal health is an ethical imperative, as the health of our nation depends on the most vulnerable populations being adequately protected against disease [18]. To obtain such a state, action must be taken to address historical injustices and eliminate health disparities such as obesity, a preventable disease [19]. 

To develop effective interventions for under-represented youth, cultural factors need to be considered to help ensure the appropriateness and relevance of program content and structure [14,20]. This includes an awareness of broadly shared cultural values, beliefs and expectations [21]. Community-engaged research, i.e., partnering with the community of interest, to develop program content and structure [22,23], is one way to help ensure a program is culturally aware and reflects important characteristics of the community, including their needs, interests and expectations. Community-engaged research also provides an opportunity for co-learning between the community of focus and researchers, thus increasing the likelihood of more equitable outcomes and stronger, more relatable interventions [24,25].

The Butterfly Girls program (BFG) was designed to address the inequities in obesity prevalence among Black girls. The program was developed using a community-engaged approach with the Black community in the greater Houston, TX area. The purpose of this paper is to report parent and child reactions to the program. 

## 2. Materials and Methods

### 2.1. Intervention 

Although described in detail elsewhere [26], the program will be briefly described here. The BFG program was a three-group randomized controlled trial, consisting of a treatment, comparison and waitlist control group. Data were collected at three timepoints: baseline (prior to receiving the intervention), post 1 (after intervention completion—i.e., approximately 3 months post baseline); and post 2 (approximately 6 months post baseline). The eight-episode online intervention promoted five servings of fruits and vegetables, five glasses of water and 60 min of physical activity per day. Both girls and one parent received an intervention. The centerpiece of the girls’ program was animated stories, populated with six characters designed to serve as role models; the stories were supported by goal setting, goal tracking and feedback. Parents received electronic newsletters corresponding to each episode the girls viewed. Girls could view one episode each week. They received an automatic reminder email when they were eligible to view the next episode and the parents received an email with a link to the episode-specific newsletter. For participants who did not view an episode within three days of eligibility, an email reminder was sent. A reminder call was made to the parent after six days and a follow-up call was made every five days until the child completed the episode (up to five calls); the program was structured so that the girls did not miss an episode (i.e., episodes were available sequentially, regardless of the time lapse between logins). Girls randomized to the treatment group received the full intervention immediately after completing baseline data collection (i.e., animated stories, goal setting, goal tracking and feedback); those in the comparison group (i.e., animated stories only) also received the animated stories after completing baseline; and those randomized to the waitlist control group received the treatment intervention without reminder emails after completing all three data collection timepoints (baseline, post 1, post 2). 

The BFG program was adapted from an earlier pilot intervention consisting of a summer day camp, followed by an internet component to promote maintenance of change [27]. The internet component was later tested as a stand-alone internet intervention [10].

During adaptation of the BFG intervention [26], community-engaged research was conducted with three groups of stakeholders to provide feedback on the cultural and developmental appropriateness of program content and graphics, as well as to develop a deeper understanding of expectations. The panels consisted of girls (n = 20), parents (n = 20) and community representatives (n = 10). The girls and parents participated in interviews and the community representatives completed online surveys with similar questions to the interviews. Trained staff conducted the interviews following a semi-structured script; each interview lasted about 1.5 h. To help ensure cultural appropriateness and relevance, a Black female playwright authored the scripts for the animated stories. 

### 2.2. Eligibility Criteria

Black girls, 8–10 years old living in the greater Houston, TX area, were recruited using standard procedures and the volunteer database at the Children’s Nutrition Research Center. Eligibility criteria included 8–10-year-old Black girls with a personal email address, internet access and a parent or legal guardian who was willing to allow them to participate and would themselves participate in the parent component of the program [26]. While girls needed to self-identify as Black to be eligible for participation, parents were not required to identify as Black. 

### 2.3. Data Sources

This paper reports parent and child reactions to, and child participation in, an intervention designed using community-engaged research. Data for this paper included parent and child quantitative and qualitative data from baseline and post 1 (immediate post-intervention) data collection time points. Surveys were completed online using a private password. As part of baseline data collection, parents completed demographic surveys describing personal and home characteristics. The characteristics included total household income, highest level of household education, parent race and ethnicity, parent gender and the number of adults living in the household including themselves. At post 1 (immediately after their daughter completed the intervention), parents of girls randomized to the treatment or comparison group were eligible to complete a process evaluation survey. The survey included two open-ended questions. The first question required a yes/no response: “Would you recommend this program to other parents?” followed by, “Please tell us your reasons for recommending or not recommending the program to other parents”. At the end of the survey, parents were given an opportunity to provide additional information: “Please use this box for any additional comments you may have”. For the girls, program participation data was automatically collected in each session as girls logged in and interacted with the program. At post 1, girls randomized to the treatment or comparison group were eligible to complete a process evaluation survey with one open-ended question: “If there is anything you would like to tell us about the characters, their voices, the story, or the program, please type it in here”. 

### 2.4. Quantitative Analysis

Survey responses were analyzed using descriptive statistics (frequencies, percentages). 

### 2.5. Qualitative Analysis

A structured analytic approach was used to code parent and child data [28]. Prior to analysis, codebooks containing a priori codes and definitions were developed and agreed upon by coders. Separate codebooks were developed for parent and child comments. The parent codebook included six a priori codes: benefits, compliments, suggestions, barriers, data collection and requests. The same codebook was applied to all parent comments reported in this paper. Similarly, the child codebook contained five a priori codes: compliments, relatability, complaints, requests and benefits. Two coders independently applied the codebooks to parent and child comments for each question. Coding was then compared. Differences were discussed and resolved by mutual agreement. After differences were resolved, for both the parent and child codebooks codes were examined and converted to categories. Comments within categories were examined for similarities in underlying topics or ideas, then grouped into subcategories. 

## 3. Results

### 3.1. Household and Demographic Characteristics

Three hundred thirty parents completed the demographic survey (Table 1). Nearly all of the parents identified as Black (93.0%) and female (98.8%). Most were less than 40 years old (59.4%), married (62.7%) and lived in households in which the majority (70.3%) lived with one other adult or by themselves. Household income was nearly evenly split between those making $41,000 or less per year (43.0%) and those making $61,000 or more a year (38.8%). Most reported that the highest level of household education was a college degree or greater (65.4%).

### 3.2. Parent Results

Parents (n = 205) responded to the survey question as to whether or not they would recommend the program to other parents and provided reasons for such a decision. Overall, 98.1% of the parents said that they would recommend the program to other parents. 

Of these, 184 parents responded to the question, “Please tell us your reasons for recommending or not recommending the program to other parents” and 65 parents responded to “Please use this box for any additional comments you may have”. Responses to both questions were separately coded and grouped into six categories: benefits, compliments, suggestions, barriers, data collection and requests. Results are summarized by question below, supported by representative comments to provide context and insight. Verbatim comments are shown to provide additional insight. To ensure categories accurately captured parent comments, responses could be coded into multiple categories, depending on response content. 

**Figure 1:**
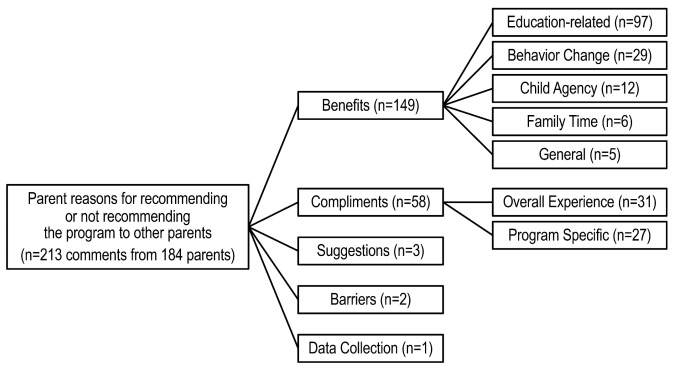
Reasons parents would or would not recommend the Butterfly Girls intervention.

For the question: “Please tell us your reasons for recommending or not recommending the program to other parents”, 213 comments were grouped into five categories (Figure 1). The category with the most responses was benefits, receiving a total of 149 comments. Comments related to benefits were grouped into 5 subcategories: education-related (n = 97), behavior change (n = 29), child agency (n = 12), family time (n = 6) and general (n = 5). 

The majority of the comments noted the education-related benefits of the program. For instance, parents often stated that the program added “educational value to both parents and kids on living healthily” or generally described it as informational, using words like “informative” and “great information” or made the parents aware of their current dietary habits by using phrases such as “it [the program] helps me and my daughter realize what we ate”. 

Comments on behavior change indicated that the program was motivational and encouraged healthy eating and physical activity. One parent noted that the program provided “great motivation for healthy changes”. Another parent said, “this program is [a] good way to encourage girls to get and stay active”. 

Child agency was defined as enhancing self-confidence regarding health choices. For instance, one parent said the program will help “children make better choices for themselves and encourage their friends”. Another said it helps “girls have a positive body image”. 

Comments related to family time often focused on the program being a bonding opportunity as indicated in this comment: “this program brings mothers and daughters closer”. Another parent said the program helped them “think about the type of foods I was purchasing for my family and some ways we could improve as it relates to eating and just spending quality/active time with the kids”, indicating that the program itself was a bonding experience and helped encourage bonding outside of the time spent on the program. 

Responses coded under general were not specific enough to be subcategorized. They typically stated something like “great program for young girls” or that “other parents can benefit from this program”. 

Compliments contained the second largest category of responses (n = 58). These responses were further sub-categorized as compliments about their overall experience (n = 31) and program-specific compliments (n = 27). Responses that were complimentary of the overall experience indicated that parents felt positive about their time in the program. One parent indicated that it was a “great experience for [their) child] and good interaction between parent and child”. Another parent described their overall experience as “relevant to issues in our society with African Americans, engaging and convenient”. Several of the comments described their experience in BFG as being engaging or “fun”. 

Program-specific comments tended to focus on the information related to health and nutrition provided by the program. For example, a parent said, “I find the program very informative and kid-friendly. The language use is clear and precise and on the level that children understand”. 

Few comments (n = 3) were coded as suggestions. One parent indicated that they thought the program would be better if children were asked to watch the videos and repeat the information to the parent in their own words rather than having parents watch the videos with them. They thought the program could be turned into a reading and writing assignment to “sharpen critical thinking skills”. Another parent wished the program had been offered in-person. 

Similarly, few comments (n = 2) were coded as barriers. Both were time-related. One parent said, “great program, but time consuming”, while the other said the program took “a lot of time and effort”. 

Only one comment focused on the data collection experience. This comment described the parent’s awareness of the importance of data collection for healthy lifestyles. 

There were no comments coded as requests, which were defined as asking for consideration of some type or changes they would like to see in future iterations of the program. 

**Figure 2:**
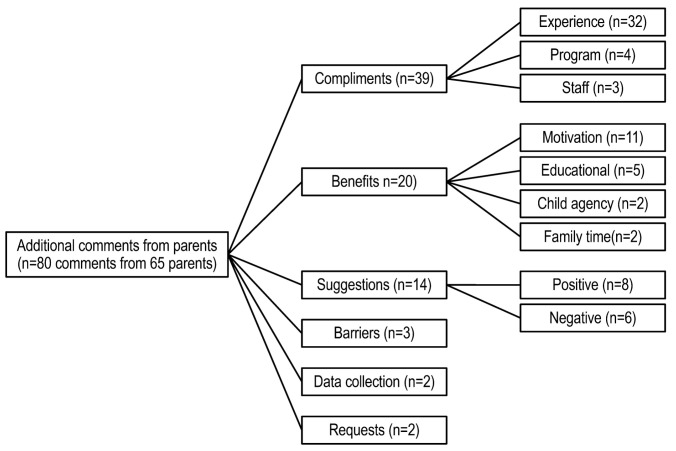
Parent comments on the Butterfly Girls intervention.

Parents were also given an opportunity to respond to the question “Is there anything else about this program that you would like us to know”. Responses from 65 parents were grouped into six categories, listed in descending order: compliments, benefits, suggestions, barriers, data collection and requests *(*Figure 2).

The majority of the responses to the question were coded as compliments (n = 39). Compliments were further sub-coded as compliments on the overall experience (n = 32), program (n = 4), or staff (n = 3). Compliments on the overall experience tended to be general, e.g., one parent said “it’s a wonderful program”. Compliments on the program focused on how “interactive” parents found it or on the “ideals” exemplified in the episodes. Comments on the staff described their interactions as positive and found it helpful to have reminders from the research team to complete episodes.

Parents also viewed the program as beneficial (n = 20 comments), further grouped into four subcategories: motivation for behavior change (n = 11), educational (n = 5), child agency (n = 2) and family time (n = 2). The majority of the comments were related to behavior change. For example, many of the comments given by parents were similar to this statement: “the program helped us reinforce what we’re trying to do as a family”. One parent said that they added more fruits and vegetables to their meals and enrolled the children in physical activity programs following program completion. Additionally, parents commented on the educational aspects of the program saying that they learned more about “good food habits” and considered the program to be a resource. Agency was mentioned by two parents. For example, one noted that her daughter is “very proud about her part in the study… It is now great to see how she compares what she has learned from the videos…to life as she sees it and is able to see how either herself or other(s) can make better choices”. Family time was also mentioned by a few parents. As one parent commented: the program “opened up a dialog between the two of us (mother and daughter) about changes we could and should make to our families’ diet.”

Fourteen responses were coded as suggestions. Suggestions were further subcategorized as positive (n = 8) or negative (n = 6). Several of the positive responses called for expansion of the program in some way: for example, two parents suggested expanding the age range and one suggested targeting the program to low-income families. Negative responses primarily focused on the videos, ranging from parents asking to expand the videos in order to demonstrate more ways to add vegetables into diet, to the need for better technical quality and more realistic character voices. Shortening the time commitment needed to complete the dietary recalls was also suggested.

Very few comments (n = 3) focused on barriers. Two parents indicated that they did not have sufficient time to incorporate more of the lifestyle changes they had wished to incorporate, while another indicated they faced a financial barrier to buying produce. Finally, one parent indicated that they were unable to get the rest of the family interested in making lifestyle changes suggested in the videos. 

Two responses were coded under data collection. Both were coded as negative. One parent indicated that the diet recall survey “wore out” her daughter due to issues with attention span (i.e., 24-h dietitian-assisted recall). The other parent indicated that the activity tracker was uncomfortable for her daughter to wear (i.e., Actigraph monitor was worn for 7 days at each data collection period). 

Two comments were coded as requests. One parent requested a focus on promoting independence such as including an activity where the child shops for recipe ingredients. Another parent indicated a desire to have similar programming for a son. 

### 3.3. Child Results

After completing baseline data collection, 342 girls were randomized to condition: 114 to the treatment (full intervention) condition, 114 to the comparison condition (animated stories only) and 114 to the waitlist control condition. Episode completion for all groups was automatically cataloged as girls navigated the online program. The majority of girls assigned to the treatment (53.5%) and comparison (68.4%) conditions watched all 10 episodes, while few girls in the waitlist control condition did so (4.4%) (Table 2). 

**Figure 3:**
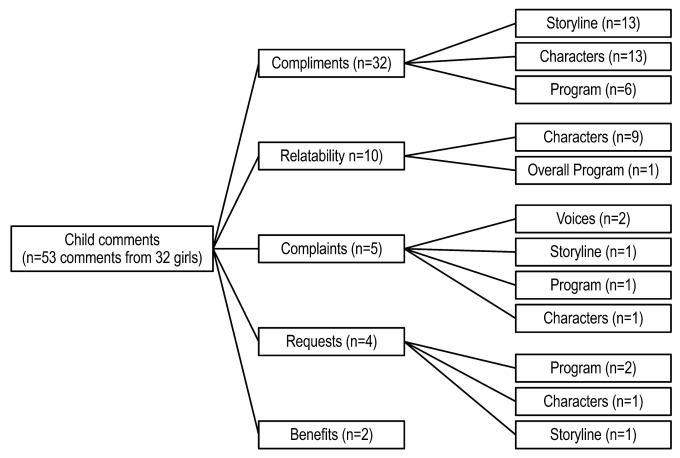
Child comments on the Butterfly Girls intervention.

Girls (n = 32) provided responses for the question “if there is anything you would like to tell us about the characters, their voices, the story, or the program, please type it in here”). Responses were grouped into five categories: compliments, relatability, complaints, requests and benefits. Results for each category are summarized below and in Figure 3, supported by representative comments to provide additional insight. As with parents, to ensure categories accurately captured child comments, responses could be coded into multiple categories, depending on response content. A total of 53 comments were coded.

The category receiving the most comments was compliments (n = 32). Comments were grouped into three subcategories: storyline (n = 13 comments), characters (n = 13 comments) and overall program (n = 6 comments). In general, girls felt positive toward the storyline. One girl liked how the story was put together and another found the plot “easy to follow along with”. Another girl simply said, “I love everything about the stories”. Compliments regarding characters generally focused on positive attributes they admired in the characters such as strength, confidence, or athleticism. For instance, one girl described the characters as “strong, confident, peaceful and more”. Several girls expressed positive reactions to the overall program, most frequently focusing on the fun they had while participating in the program. For example, one girl commented that the program was “fun, creative and easy to read”. 

Ten comments from nine girls were coded as relatability, defined as the child feeling positively represented by the story, feeling the overall experience was inclusive, or feeling represented by the characters. Comments were further grouped into reactions to the characters (n = 9) and the overall program (n = 1). Findings indicate that girls felt connected to the characters, particularly those who shared a common interest with the girls and looked like them. For example, one girl stated, “I like the characters because they relate to me because I like butterflies and fashion”, while another said, “[the girl characters] remind me of my friends and cousins”. Finally, another indicated that she related to the program saying that participating in the program made her feel part of a “special group”. 

Five comments were coded as complaints. The comments were further grouped into reactions to the voices (n = 2), storyline (n = 1), program (n = 1) and characters (n = 1). Findings were varied. Two girls found the voices “weird”. One girl wished the girls had been shown going to school. Another girl had technical difficulties in finishing an episode. Lastly, one girl indicated disliking the character who “berated her friends”. 

Four comments were coded as requests. These comments related to reactions to the program (n = 2), characters (n = 1), or storyline (n = 1). Requests were varied. One girl wished to taste the recipes she did not get to try during the program. Another girl indicated a desire to repeat the program with a different story stating that she would “love to do it again with a different story”. Regarding the comment related to characters, one girl wished for a change to a character’s role in the storyline requesting that they played for a different soccer team. Similarly, one girl indicated a desire for nicer antagonists (in the storyline, boys were competing with the girls to locate Founder’s Rock). 

Lastly, two comments were coded as program benefits. One girl said, “it was great how the butterfly girls taught me to stay active”. The other girl stated that the program gave her the opportunity to interact with computers.

## 4. Discussion

Parent/child pairs (n = 342) participated in the Butterfly Girls program promoting healthy diet and physical activity behaviors for 8–10-year-old Black girls. The program was developed using community-engaged research with multiple stakeholder groups, including 8–10-year-old Black girls, their parents and community representatives. Girls randomized to the treatment or comparison groups demonstrated high levels of program participation. This study aimed to understand parent and child reactions to the program following community engagement during program development. Future papers will utilize machine learning to detect patterns in diet, physical activity and body weight change following the BFG program. Parent and child comments indicated high connectedness to the program and that it was viewed as an educational and motivational resource. Results also provide evidence that a program designed with key stakeholders from the community for whom the intervention is intended can result in high program participation. This suggests that co-design approaches, such as community-engaged research, are essential for programs designed to attain health equity.

The demographics of the study sample were socioeconomically diverse with 43.0% of parents earning $41,000 a year or less and 38.8% of parents earning over $61,000 a year. However, the majority of the households represented had an individual with at least a college degree. The fact that education status did not necessarily predict the socioeconomic diversity of the sample is consistent with other studies in minority populations that have shown that income disparities can persist despite educational status [29,30]. 

Program dose was relatively high with 68.4% of the girls in the comparison group and 53.5% in the experimental group watching all of the BFG episodes, suggesting an acceptable program dose. Alternatively, girls in the wait-list control group watched few episodes. A community-based obesity prevention program for parents and children with longitudinal interactions with research staff reported similar attendance to those in our treatment and comparison conditions, with an average attendance of 64.0% of sessions [31].

Striking differences in program dose were observed among the groups, which may partially be explained by participation reminders. While girls in the treatment and comparison groups received reminders to log on, those in the waitlist control group did not. Girls in the treatment and waitlist control groups received the same intervention, with the only difference being participation reminders, suggesting that reminders can enhance participation. The literature supports the importance of participation reminders in program adherence. Shumaker et al. [32] and Robiner [33] emphasized the importance of staff in program adherence. 

The qualitative findings from the girls indicated connectedness to the characters and positive perception of the overall storyline and characters. These findings support the importance of community-engaged research when developing interventions for under-represented children. Collaboration with minority populations in the research process allows for the cultural sensitivity the girls’ comments highlighted [21]. The girls’ positive feedback suggests that collaboration with Black girls in the program development strengthened the program and may have facilitated their high levels of participation. BFG may have been less well-received or adherence may have been lower had cultural factors not been considered in the storyline or character development. Evaluation of youth programming suggests that including their participation throughout program development and implementation can result in greater connection with program messages [21,34]. Other studies with minority populations support the importance of cultural sensitivity in intervention development. Specifically, a study with Black girls requested Black female role models in programming and at-home strategies for physical activity and healthy eating promotion [14]. 

The qualitative responses from the parents indicated that they found the program to be beneficial, primarily as an educational resource and secondly as a motivator for behavior change, and was an overall positive experience. Parental perception is of particular importance given that parents can be a powerful determinant of children’s health behaviors [35,36]. Parents’ perspectives on strategies for healthy dietary choice promotion for children have been well studied [37,38]. However, less attention has been given to minority parents’ perceptions regarding ways to help children practice healthy lifestyle behaviors [29,39,40]. Furthermore, community-based participatory research, while it can be difficult to evaluate its effect on participation, shows that participant engagement strengthens the program’s ability to equitably distribute the benefits of a program or overcome barriers such as in our socioeconomically diverse parent population [21,41]. Parent perception of other obesity prevention programs is in line with what the parents in the BFG study perceived as beneficial. Specifically, Black parents in a text messaging-based study suggested a need for resources on tips for helping their daughter make healthy dietary choices [42]. 

The BFG Program had several limitations. Perhaps in part due to the developmental maturity of participants, only 32 children responded to the qualitative prompts in the Post 1 timepoint survey. Furthermore, there is a possibility that responses for parents and children to qualitative prompts were self-selecting in that only those who had significant experiences in the BFG program gave an answer. Further, parental educational status was relatively high, which may limit generalizability. However, the socioeconomic status of parent participants was diverse which may lessen this concern. Finally, it is possible that the Hawthorne effect influenced participation given that participants received phone calls from researchers if they failed to watch an episode [43]. Despite its limitations, the BFG program was strengthened by its three-group design that included a wait-list control group and a comparison group that did not receive the behavioral goal-setting components. In addition, the use of automatic recording of session completion avoided errors due to self-reporting. Finally, the BFG program was strengthened by the socioeconomic diversity of the participant sample. While 43.0% of participants lived in a household with a total income of $41,000 or less, 38.8% lived in households with a total annual income of greater than $61,000, demonstrating the economic diversity and its generalizability. However, future studies should explore programming specific to certain socioeconomic groups, as minority populations are not homogenous. 

## 5. Conclusions

This study provides evidence that a co-design approach via collaboration with key community stakeholders can encourage program engagement, as evidenced by high program participation rates and positive reactions to the program. These findings suggest that a co-design approach is an important component of programs designed to achieve health equity. 

## Figures and Tables

**Table 1:** Parent Demographics and the Home Environment (n = 330).

Characteristic	Subcategory	Percentage of Total—330 (%)
Age		
	≤39 years old	196 (59.4%)
	>40 years old	134 (40.6%)
Parent race		
	Black	307 (93.0%)
	White	10 (3.0%)
	Black-Mixed	8 (2.4%)
	Other	5 (1.5%)
Parent ethnicity		
	Hispanic	11 (3.3%)
	Not Hispanic	319 (96.7%)
Marital status		
	Married	207 (62.7%)
	Single	52 (15.8%)
	Divorced, widowed	71 (21.5%)
Gender		
	Male	4 (1.2%)
	Female	326 (98.8%)
Average annual household income		
	<$21,000	43 (13.0%)
	$21,000–$41,000	99 (30.0%)
	$42,000–$61,000	60 (18.2%)
	>$61,000	128 (38.8%)
Highest level of household education		
	Some high school	2 (0.6%)
	High school/GED	13 (3.9%)
	Technical School	8 (2.4%)
	Some college	91 (27.6%)
	College graduate	113 (34.2%)
	Postgraduate study	103 (31.2%)
Total adults living in home (including respondent)		
	≤2	232 (70.3%)
	≥3	96 (29.1%)
	Missing	2 (0.6%)
Children <18 years old living in home		
	≤2	193 (58.5%)
	≥3	137 (41.5%)

**Table 2:** Episodes Children Watched (n = 114 per group; percentage reported per group).

	All Episodes Watched	Some Episodes Watched	No Episodes Watched
Group 1	61 (53.5%)	42 (36.8%)	11 (9.6%)
Group 2	78 (68.4%)	30 (26.3%)	6 (5.3%)
Group 3	5 (4.4%)	28 (24.6%)	81 (71.1%)

## Data Availability

The datasets generated and/or analyzed during the current study are not publicly available due to concerns regarding privacy but select data are available from the corresponding author on reasonable request.
